# Near-infrared photons: a non-invasive probe for studying bone blood flow regulation in humans

**DOI:** 10.1186/s40101-015-0066-2

**Published:** 2015-07-25

**Authors:** Tiziano Binzoni, Lorenzo Spinelli

**Affiliations:** Département de Neurosciences Fondamentales, University of Geneva, Geneva, Switzerland; Département de l’Imagerie et des Sciences de l’Information Médicale, University Hospital, Geneva, Switzerland; Istituto di Fotonica e Nanotecnologie, Consiglio Nazionale delle Ricerche, Milano, Italy

**Keywords:** Bone blood flow regulation, Human, Bone neurovascular system, Blood volume regulation, Near infrared light, Biophotonics

## Abstract

The study of bone blood flow regulation in humans has always represented a difficult task for the clinician and the researcher. Classical measurement techniques imply the presence of ionizing radiation or contrast agents, or they are slow or cannot be repeated too often in time. In the present review, we would like to give a perspective on how the optical approach might overcome some of these problems and give unique solutions to the study of bone blood flow regulation. We hope that the present contribution will encourage the scientific community to put a greater attention on this approach.

## Introduction

The study of bone blood flow regulation in humans has always been a challenging topic. In fact, known techniques allowing the monitoring of blood flow appear to be invasive (e.g., utilization of ionizing radiation or contrast agents) or do not permit fast measurements (e.g., to follow blood flow pulsations) over repeated or long periods of time. Moreover, these techniques are often very expensive and cannot be used at the patient bedside [1–4]. In this frame, optical technologies, based on near infrared light, might represent a unique solution allowing to surmount some of the these difficulties and open the possibility to approach from a new angle the study of blood flow regulation in bone.

Light in the 650- to 950-nm range has the singular property to deeply penetrate biological tissues such as brain, muscle, fat, or bone. Moreover, due to the phenomenon of diffusion, light propagates in all directions, and then, it is re-emitted also from the same surface where it is injected. Photons composing this light interact in various ways with living tissues, and when detected, they carry information about blood flow and other parameters related to it. In the following sections, we will explain, by reporting the related scientific literature, how non-invasive and contrast-agent free optical techniques can be exploited to investigate the regulatory mechanisms of the bone neurovascular system in humans.

## The optical instrumentation

**Fig. 1:**
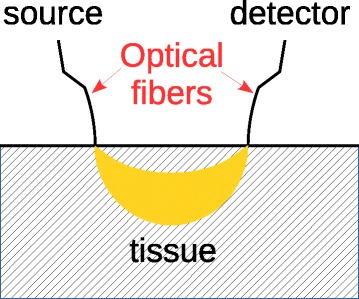
Monitored region of interest. Intuitive drawing representing the “banana shape” region of interest investigated by the different optical instruments presented in the manuscript. The larger the source/detector spacing is, the deeper goes the minimum of the “banana”, and thus, a deeper region can be investigated.

For the present purposes, all the instrumentation of interest can be intuitively seen as a clever combination of a light source and a photodetector. The light is usually transported to the tissue by an optical fiber in contact with the skin. The light diffuses into the underlying tissue and is collected a few centimeters apart from the injection point by a second optical fiber (or a bundle of optical fibers), which delivers it to a photodetector. Thus, the optical fibers need not to be inserted in the probed tissues, but they lie gently, normal to the skin surface. It is common to say that, as a first approximation, the region of interest investigated by these instruments has a “banana” shape; the extremities of the “banana” being localized on the two fibers tips (see Fig. 1). Obviously, other geometrical configurations can be chosen depending on the experimental purposes. However, considering that all the literature cited in the present mini-review adopt a “banana”-shaped configuration, other geometries will not be treated. From Fig. 1, one can intuitively observe that the larger is the inter-fibers spacing, the deeper the light will penetrate and thus deeper tissues will be monitored (e.g., bone under the skin/fat layers). This allows, e.g., to obtain “mean” penetration depths of 1–2 cm (the maximum depth is larger). By choosing different types/combinations of light source(s) and detector(s), different optical instruments allowing to investigate various bone blood flow related parameters may be created. Light sources may be for example light emitting diodes (LED), continuous wave (CW) or pulsed lasers, or white light emitting bulbs. Photodetectors may be, for example, avalanche photodetectors, photomultipliers, or single-photon detectors. Coming back to the penetration depth issue, it is worth to note that large inter-fibers spacing requires more powerful sources and/or sensitive detectors. Thus, independently of the chosen approach, different mean depths may be reached, also depending on the quality of the source/detector hardware that has been employed for the actual implementation. Finally, from Fig. 1, one can infer that in general, due to the presence of the optical fibers, we are dealing with a “contact” (but non-invasive) instrumentation, investigating large tissue volumes. All the optical techniques presented in this review are contrast-agent free.

Table 1 reports the summary of the typical optical instrumentation, with the main technical characteristics, that will be considered in the following sections. In Table 1 are also reported the main physiological parameters measured by the different instruments.

**Table 1:** Optical instruments utilized to investigate bone blood flow in humans

Instrument name	Possible source types	Possible number of light wavelengths	Light source intensity	Detected parameters for light (possible detector types)	Measured physiological parameters
PPG [32]	LED	1	Constant	Intensity (PMT, APD)	*Δ*BF (a.u.)
NIRS [33]	LED, CW laser, white light	1, 2, 3, …, to continuous	Constant	Intensity (PMT, APD)	Hb, HbO_2_, *Δ*Hb, *Δ*HbO_2_, (a.u.) and %SO_2_
DCS [34]	CW laser	1	Constant	Temporal correlation (SPD)	BF (cm^2^ s ^−1^)
IMS [35]	IM laser	2, 3, …	Sinusoidal modulation	Intensity and intensity phase shifts (PMT, APD)	Hb, HbO_2_, (*μ*M) and %SO_2_
TRS [36]	Pulsed laser	2, 3, …	Pulsed	Photon DTOF (SPD)	Hb, HbO_2_, (*μ*M) and %SO_2_
LDF [37]	CW laser	1	Constant	Doppler frequency shift (PMT, APD, SPD)	BF, BS, #rbc (a.u.)

Note that concentrations are relative to the volume of the investigated region of interest. For the present purposes the source-detector geometrical configuration is the same for all the instruments and is schematically represented in Fig. 1. The bibliographic references appearing here are technical references.

*PPG* photoplethysmography, *NIRS* near infrared spectroscopy, *DCS* diffuse correlation spectroscopy, *IMS* intensity modulated spectroscopy, *TRS* time resolved spectroscopy, *LDF* laser-Doppler flowmetry, *LED* light emitting diode, *CW* continuous wave, *IM* intensity modulated, *DTOF* distribution of time-of-flight, *BF* mean blood flow, *Δ* *BF* change in BF, *Hb* deoxy-hemoglobin concentration, *H* *b* *O*_2_ oxy-hemoglobin concentration, *Δ*[*H* *b* change in Hb, *Δ*]*H* *b* *O*_2_, change in *H* *b* *O*_2_, % *S* *O*_2_ blood oxygen saturation, *BS* mean blood speed, *#rbc* number moving red blood cells, *PMT* photo-multiplier tube, *APD* avalanche photodetector, *SPD* single-photon detector, *a.u.* arbitrary units

The aim of the present contribution is to show to a non-technical reader the potentialities of the different optical instrumentation when applied to the study of human bone blood flow. For this reason, we will not discuss technical or theoretical advantages/problems related to the different approaches, because this is out of scope. The reader interested on a more technical side of this presentation can refer to specialized literature [5, 6].

## Investigations on bone blood flow by the optical approach

For this section, an organization per instrument has been conceived, emphasizing what the different optical techniques can actually measure, in the framework of human bone blood flow investigations. As a matter of fact, the specific parameters of interest for the investigator directly depend on the type of optical instrument. We think that this approach is closer to the real needs of the experimenter or the clinicians that first of all wants to know what it is possible or not to measure with the optical approach, without being lost in technical details or often confusing chronological history.

### Near infrared spectroscopy

Near infrared spectroscopy (NIRS) allows the assessment of oxy-(HbO_2_) and deoxy-hemoglobin (Hb) concentration changes (*Δ*HbO_2_ and *Δ*Hb, respectively). This is made possible by the fact that light is absorbed differently by HbO_2_ compared to Hb. Some clever mathematical algorithm, exploiting this phenomenon, allows one to obtain *Δ*HbO_2_ and *Δ*Hb as instrument output.

An early attempt to monitor non-invasively bone blood perfusion, by optical techniques, has been performed by NIRS in human tibia [7]. The aim was to define an indirect perfusion index allowing future investigations on blood flow regulation in special environmental conditions such as in microgravity. The rates of deoxy-hemoglobin concentration changes were utilized as an index allowing to evaluate the reperfusion speed after a short ischemic period. It was shown (13 males) that the return to preischemic conditions is faster for the tibialis muscle than for the tibia (from ∼1.8 times at 72 years old to ∼3.5 times at 25 years old) and that this kinetic becomes slower with aging in both tissues (∼2 times for tibia from 25 to 72 year old). In a subsequent NIRS study [8], these results have been confirmed (13 males) and it was also shown that, in tibia bone, resting blood oxygen saturation (%SO_2_) remains constant (∼85 %) with aging, while in tibialis muscle, it decreases (−0.35 % per year with an %SO_2_ of ∼80 % at 25 years old). This gives us some inside on the age dependence of bone vascular system and related oxydative metabolism, information that cannot be easily obtained by other classical measurement techniques. Always using NIRS, it has been shown that, as expected, tibia oxygen consumption does not change during submaximal (80 %) isometric contractions (foot dorsiflexion, 10 males and 5 females) [9]. On the other side, despite the rigid bone structure, NIRS has allowed to demonstrate that orthostatic stress (tilt bed) may induce blood volume changes in human tibia bone/bone marrow [10] (6 males). This phenomenon is probably related to a concurrent extravascular fluid displacement in the tibia and a typical half time of 1 min (estimated from Figure two in [10]) is necessary to reach a *Δ*Hb + *Δ*HbO_2_ steady state level after tilting. This value is independent of the magnitude of the rapid increase in the orthostatic pressure. In the same study, a sudden change (at ∼28 mmHg) in the slopes of *Δ*Hb and *Δ*HbO_2_, as function of the orthostatic pressure, was also revealed. This was interpreted as the consequence of the possible presence of a direct and/or indirect venous-arteriolar reflex. Since then, NIRS instruments specifically dedicated to bone investigation have been developed [11]. This has allowed to better define the region of interest monitored by the NIRS instruments and, as a consequence, by any other optical instrument presented here (thanks to the similarity in the optical geometries; see, e.g., Fig. 1).

Attempts to apply NIRS in clinical settings have been made on spinal cord injured (SCI, five males and five females) patients [12]. Even if it does not represent an extensive study, it has already been possible to observe, by analyzing the *Δ*Hb and kinetics, that normal subjects (five males and five females) have faster post-ischemic hyperhemic response in tibia (baseline-to-peak Hb in 10.1 s in normal compared to 16.6 s in SCI) and faster return to baseline compared to SCI [13]. The result may be explained by an impaired blood flow in SCI.

### Time resolved spectroscopy

The NIRS instrumentation considered in the previous section does not give access to the absolute values of Hb and HbO_2_ concentrations but only to their changes, often expressed in arbitrary units. This prevents the clinician to easily compare different patients. For this reason, a dedicated time resolved spectroscopy (TRS) system giving Hb, HbO_2_ in absolute values has been developed [14]. In TRS, by exploiting very short laser pulses (durations of tens of picoseconds), it is possible to measure the distribution of time-of-flight of photons arrived to the detector, after having traveled through the tissue. From these data, by means of a dedicated algorithm, one can obtain Hb and HbO_2_. By using this system, it has been shown in human calcaneous (range 25–72 years) that %SO_2_ remains constant (∼70 %) for increasing age. This is in accordance with previous NIRS findings (see above) in human tibia [8] but at a lower %SO_2_ value. The lower %SO_2_ may be explained by the different vascular structure of the two bones determining different blood flows (%SO_2_ may depend, e.g., on blood speed) and different Hb and HbO_2_ fractions. In this frame, values for Hb+HbO_2_ were found to be constant (∼23 *μ*M) in the same age range, implying that, in calcaneous, the vascular volume is independent from age.

It would be interesting to know if in general the vascular system of aging bone in healthy subjects undergoes blood flow regulatory changes. Considering the possible link existing for example between impaired bone blood flow and osteoporosis [1], this knowledge may reveal important for the comprehension of the mechanisms of initiation of this pathology. Optical techniques might be in this case a non-invasive and attractive early screening tool.

### Photoplethysmography

Photoplethysmography (PPG) has been introduced with the aim to have a more direct mean to assess blood flow, and the fundamental improvement has been the capability to obtain mean blood flow (BF) changes (*Δ*BF) in arbitrary units (a.u.). PPG technique is based on the fact that total hemoglobin concentration variations also changes light tissue absorption properties. By modeling this phenomenon, it is possible to obtain *Δ*BF. Compared to NIRS, it seems that PPG is also sensitive to blood flow speed, and for this reason, PPG should generate a signal that better represents blood flow changes. However, this potential mechanism has not yet been completely clarified and care must be taken on this point. Thanks to PPG it has been possible to monitor fast BF changes induced by heart pulsations in human patella [15] (12 males and 8 females). In a subsequent study, the same authors were able to demonstrate that the amplitude of the pulsatile BF (*Δ*BF, peak-to-peak) in patella is markedly reduced (∼[−26 %) in patient (9 males and 13 females) with patellofemoral pain syndrome when the knee is being flexed (from 20° to 90°), while in normal subjects (18 males and 15 females), *Δ*BF show no distinctive patterns [16]. This corroborate the hypothesis of a potential ischemic mechanism involved in this pathology.

Other PPG experimentation have demonstrated that in healthy humans (five males and four females), local cooling (5-min ice pack on the tibia) decreases the amplitude of the tibial *Δ*BF pulsations (∼]−33 %; from figure 3 in [17]). This might be a possible evidence of the presence of a sympathetic reflex in bone. It has also been observed that increasing local external pressure (from −50 to 30 mmHg) increases *Δ*BF pulsations amplitudes (from ∼[−3.2 to ∼1.4 a.u.) in the tibia of healthy subjects (six males and six females), with a sudden *Δ*BF decrease (∼]−0.5 a.u.) for pressure values exceeding 30 mmHg (from figure 3 in [18]). The exact reason of this behavior has not yet been clarified. To finish, it was observed always in healthy subjects that post-exercise increase in *Δ*BF pulsations in patellar bone (“hyperhemia”) depends on the intensity of the quadriceps femoris contraction [19] (28 males and 14 females). This resulted in an increase of 22 and 61 % in *Δ*BF for an isotonic exercise intensity of 60 and 75 % of one repetition maximum, respectively (duration of high was half of that of the low intensity exercise).

### Intensity-modulated spectroscopy

Intensity-modulated spectroscopy (IMS; also called frequency domain spectrometer) is based on the modulation of the amplitude of a CW laser light source. The analysis of the changes in the modulation amplitude at the output of the observed tissue region of interest allows one to estimate Hb, HbO_2_, and %SO_2_. Blood flow pulsations in patella has been indirectly monitored by IMS by observing Hb, HbO_2_, and %SO_2_ [20] variations. In this case, the authors have observed a periodic increase in Hb+HbO_2_ and %SO_2_ at each heartbeat of ∼3.1and ∼0.7 %, respectively (8 males). This result may be explained, e.g., by an increase in tissue blood volume mainly determined by the entry of the arterial blood in the tissue.

### Diffuse correlation spectroscopy

All the instrumentation cited in the previous paragraphs try to derive information on blood flow through indirect measurements or through BF changes. For this reason, diffuse correlation spectroscopy (DCS) and laser-Doppler flowmetry (LDF) have been developed to obtain measurements directly proportional to BF. Note that DCS and LDF do not allow to obtain Hb, HbO_2_, or %SO_2_. DCS is based on the fact that photons traveling through the investigated tissue undergo a frequency Doppler-shift when interacting with moving red blood cells. By analyzing the photo-electric current produced by these photons at the output of the photodetector (through an autocorrelation operation), one obtains BF. BF obtained by DCS is expressed in special cm^2^ s ^−1^ units. It has been demonstrated that the measured parameter is proportional to BF. In principle, this allows to obtain comparable values between different subjects. In fact, DCS has permitted to define a “normal” BF reference value (5.0×10^−9^ cm^2^ s ^−1^; range 4.2×10^−9^ cm^2^ s ^−1^ to 7.4×10^−9^ cm^2^ s ^−1^) for the human (15 males and 17 females) manubrium [21]; the aim of the authors is to compare in the future this BF value with that of patients with cancers.

### Laser-Doppler flowmetry

LDF directly assess BF in arbitrary units, but the supplementary advantage is that it allows to monitor two important parameters determining BF, i.e., mean blood speed (BS, in a.u.) and the number moving red blood cells (#rbc, in a.u.) [22]. As DCS, also LDF is based on the fact that photons traveling through the investigated tissue undergo a frequency Doppler-shift when interacting with moving red blood cells. By analyzing the photo-electric current produced by these photons at the output of the photodetector (through the power density spectrum assessment) one obtains BF, BS, and #rbc. Thanks to this particularity, it has been shown using ECG-gated LDF that heart-generated BF pulsations in patella, clavicle, tibial malleolus, and tibial diaphysis are determined by BS changes at constant #rbc [23] (5 males). This signifies that fast BF pulsations do not mechanically induce in this case fast blood volumes changes. ECG-gated LDF has allowed also to observe a secondary peak, in the BF pulsation, probably due to the closure of the cardiac valves. To improve the sensitivity of the LDF technique and to reach deeper tissue regions of interest, a new instrument based on single-photon counting has been developed [24]. This low-cost LDF allows to follow in real time BF kinetics in human tibia, during an ischemia-reperfusion protocol, with a time resolution of ∼1.6 s.

## Perspectives and conclusions

Compared to sophisticated imaging techniques such as MRI, PET, CT, etc, the use of NIRS, TRS, PPG, IMS, DCS, and LDF may seem very simplified approaches to the study of bone blood flow. Actually, this is a misleading point of view. Optical techniques have unique characteristics that might allow original investigations on bone blood flow regulation in humans. In fact, we must not forget that these techniques are portable, have imaging capabilities, and can be used in special environments such as during water immersion [25], in flight simulators [26], in hyperbaric chambers [27, 28], during high altitude hiking [29], in microgravity, or during parabolic flights [29, 30]. Moreover, the non-invasiveness of the approach allows us to study the bone neurovascular system without the unwanted influence of, e.g., the mental stress that might modulate the activity of the autonomous nervous system and thus potentially bias the experimental results [31]. The large spectrum of sampling rates (from fraction of second to seconds, depending on the specific implementation of a given hardware), acquisition times (from a fraction of second to hours), and the repeatability of the measurements, without incurring in potential risks determined by ionizing radiation and/or contrast agents allow exploiting optical techniques for both functional studies and monitoring purposes, enabling the conception of new investigations considered impossible until now for ethical reasons. The follow-up of acute or long term effects induced by pharmaceutical products modulating bone blood flow might also represent an interesting application.

It is difficult at this stage to decide which is the better optical technique. This question has not yet been completely answered by the scientific community. Probably, the best choice still depends on the precise experimental goal. For example, if very fast acquisition rates are needed, and only changes in BF values suffice, probably the PPG technique is superior compared to LDF or DCS. If absolute (a.u.) BF values are required, then LDF or DCS may represent the best choice. However, depending on the future technical improvements in the hardware components composing these instruments, this situation may rapidly change.

To shortly highlight some pros and cons of the proposed modalities in terms of their capability to assess bone blood flow, one can say for example that LDF or DCS are the only techniques allowing to directly obtain this parameter. PPG has the advantage of the high acquisition speed, but it can detect only changes in blood flow, and the mechanism of detection has not yet been fully clarified (living an open question on the linearity of the instrument response). NIRS, TRS, and IMS give only indirect information on blood flow, but they have the advantage to give other important physiological parameters (linked to blood flow) such as %SO_2_. However, in terms of explicit bone blood flow monitoring, NIRS, TRS, and IMS are probably the less suitable approaches.

Thus, besides the obvious potential utilization of the optical techniques for clinical purposes, the aim of the present review is to encourage orthopedists and scientist to exploit this new possibility to deepen the knowledge on bone blood flow regulation mechanisms in humans. A topic that certainly deserves the attention of the scientific and medical community.

In conclusion, the large spectrum of applications in the domain of human bone blood flow regulation, made possible by the advent of the optical techniques, potentially opens a new domain of study in physiological anthropology. In fact, nutrition, fitness, aging, sex, and growing are all parameters that may influence human bone blood flow. The access to low-cost, portable, and non-invasive optical techniques may represent in this sense a new powerful tool allowing large scale screenings and a better comprehension of long/short term bone blood flow regulation and its adaptability to human modern environment.
